# The Mystery of Multiple Masses: A Case of Anaplastic Astrocytoma

**DOI:** 10.7759/cureus.1384

**Published:** 2017-06-23

**Authors:** Pooja Sethi, Jennifer Treece, Vandana Pai, Chidinma Onweni, Zia Rahman, Siddharth Singh

**Affiliations:** 1 Cardiology, Quillen College of Medicine, East Tennessee State University; 2 Internal Medicine, Quillen College of Medicine, East Tennessee State University; 3 Cardiology, Cedar Saini Medical Center

**Keywords:** primary brain glioma, anaplastic astrocytoma, brain mass

## Abstract

Though most primary brain gliomas present as a single mass lesion in the brain, this potential diagnosis must be considered in the differential diagnosis when faced with a case of multifocal brain mass lesions. Among the most common brain tumors in humans, glioblastomas can be classified into four classes, one of which consists of anaplastic astrocytomas (AA). Due to its significant malignant potential, a prompt stereotactic brain biopsy should be considered to allow for early diagnosis. Karyotypic analysis of the specimen may allow for the discovery of 1p12q and IDH132 gene mutations. This knowledge can be used to best determine prognosis and guide therapy.

## Introduction

Intracranial lesions in adults are most often caused by metastatic disease or infection [1]. In the absence of a primary tumor found elsewhere in the body or signs of systemic infection being present, imaging studies may not provide sufficient evidence to make a diagnosis [2]. Therefore, a stereotactic biopsy of the intracranial lesion is necessary to reach a definitive diagnosis [3]. Primary brain glioma should be included in the differential of patients who present with either single or multifocal brain masses, even though they typically present as a single brain mass [4]. A brain biopsy is imperative for early diagnosis [3]. Anaplastic astrocytoma is a World Health Organization (WHO) Grade III brain glioma that is a malignant, diffusely infiltrating primary brain tumor, which usually presents as a solitary mass but can present as multiple brain lesions [5]. Gene mutations, such as 1p19q and IDH132, affect the prognosis of anaplastic astrocytoma and should, therefore, be tested to guide therapy [1-2, 5].

## Case presentation

**Figure 1:**
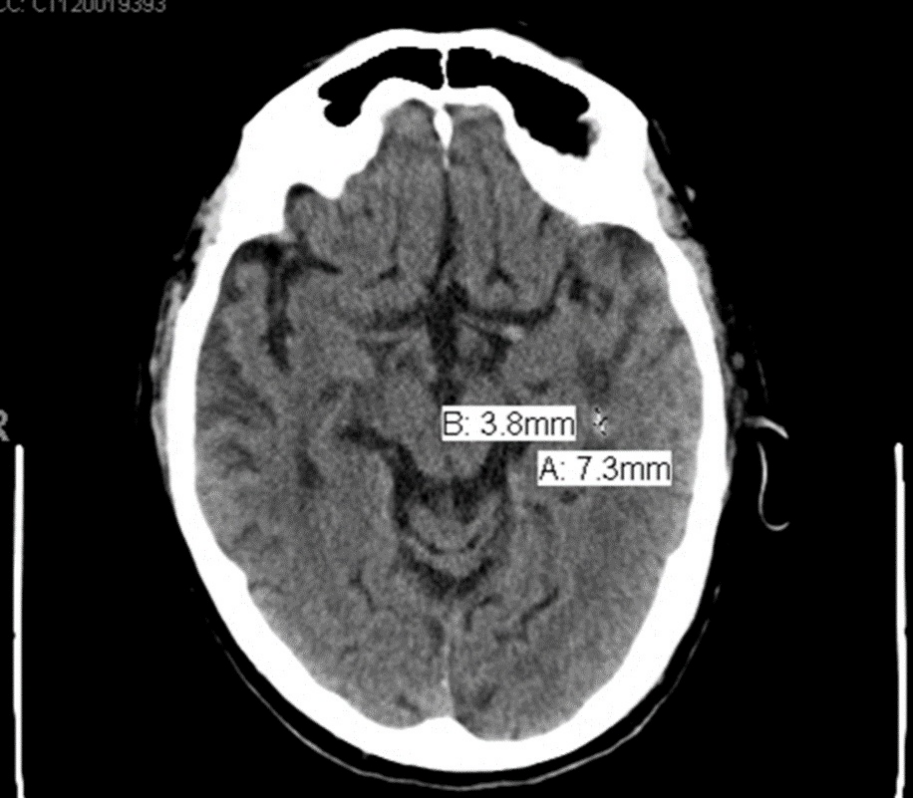
Brain masses at initial presentation. The initial computed tomography (CT) scan of the brain showed two brain masses, 3.8 mm and 7.3 mm in diameter.

A 52-year-old man presented with a two-month history of aphasia. He was evaluated by a neurologist two weeks after the onset of aphasia. An initial CT scan of his brain showed multiple, scattered brain masses, which were concerning for metastatic disease versus infection (Figure 1). A CT of his chest, abdomen, and pelvis did not reveal a primary tumor. The patient had a 30 pack-year smoking history and underwent bronchoscopy and bronchoalveolar lavage as part of his workup. Lavage fluid stained positive for Cryptococcus. A presumptive diagnosis of disseminated central nervous system (CNS) Cryptococcus fungal infection was made at that time, and the patient was started on intravenous (IV) antifungal therapy.

**Figure 2:**
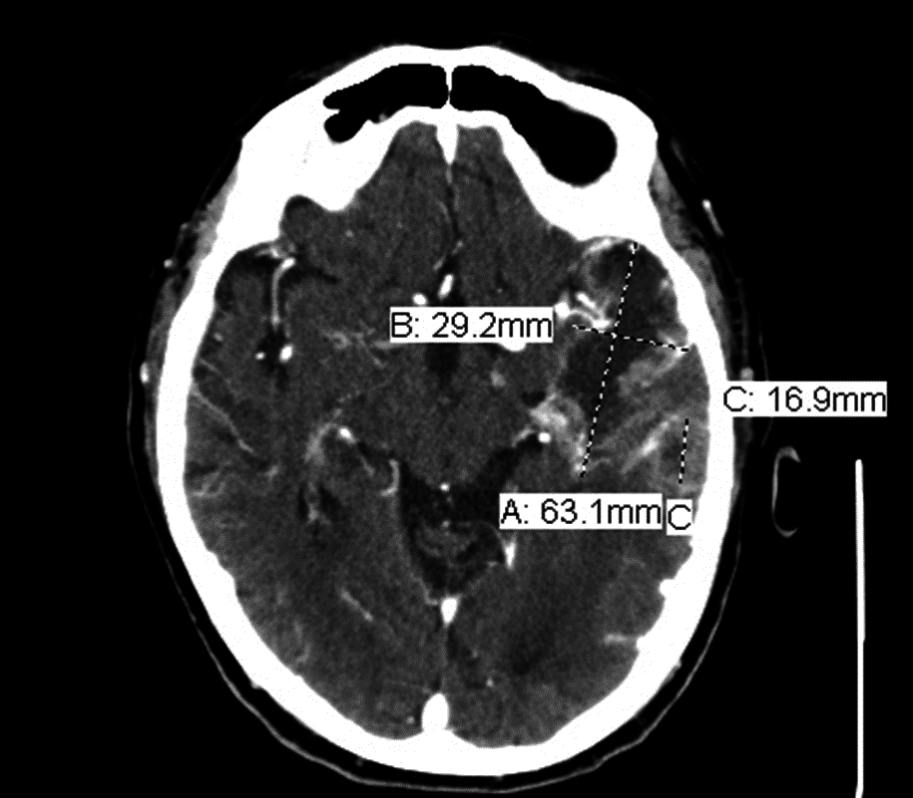
Multifocal brain masses within 2 months of initial presentation. Repeat computed tomography (CT) scan of the brain shows an increase in size of the original masses up to 63.1 mm and 29.2 mm in diameter, as well as the development of a new mass that was 16.9 mm in diameter.

**Figure 3:**
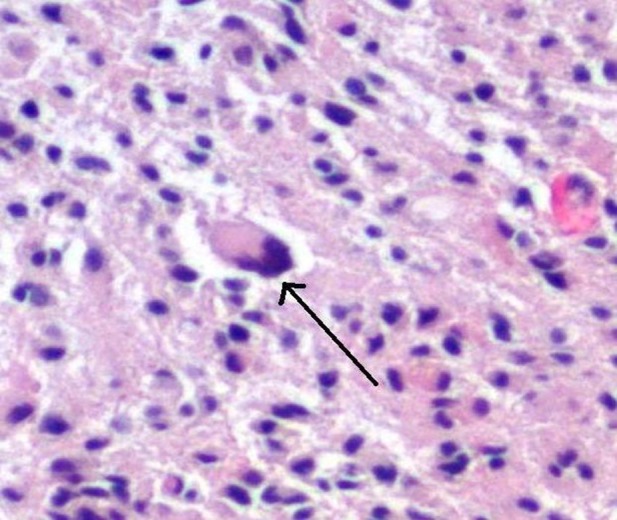
Scattered atypical cells Histopathology of the biopsied brain mass tissue shows atypical cells scattered throughout the specimen, which is indicated by the black arrow.

**Figure 4:**
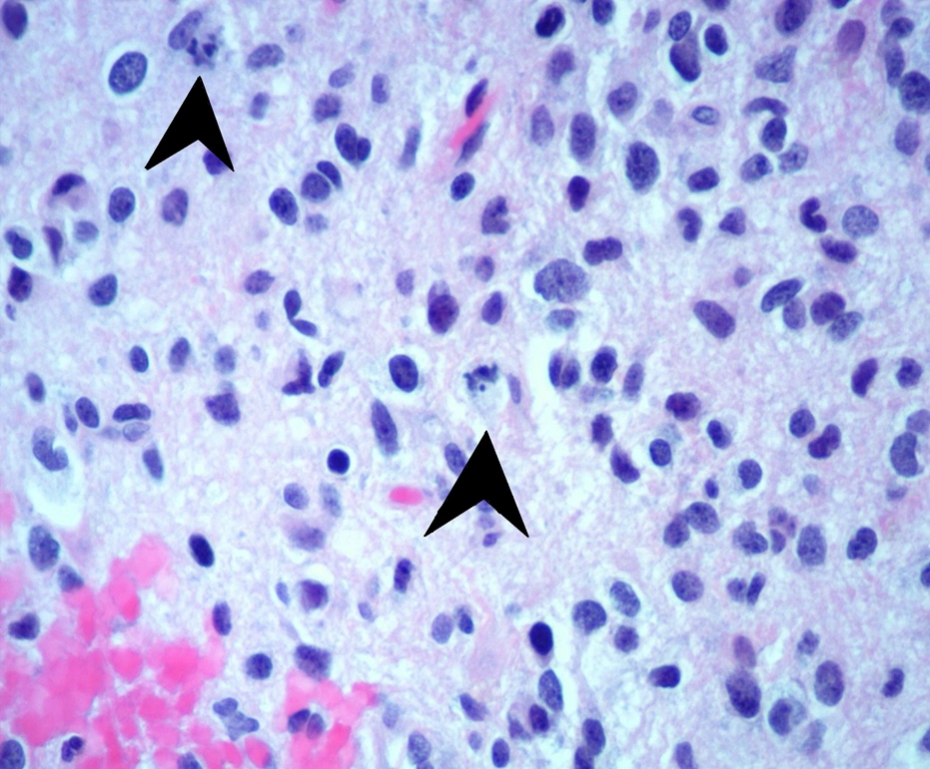
Increased mitotic activity Examination of the tissue from the biopsied brain mass shows multiple cells with increased mitotic activity, which is indicated by the arrowheads.

Despite antifungal therapy, the patient’s condition worsened, with the development of progressive limb weakness, bowel and bladder incontinence, and renal failure. A repeat CT scan two weeks later showed that the old brain lesions had increased in size, and new brain masses were detected throughout the temporal and parietal lobes as well as the cerebellum (Figure 2). He underwent a stereotactic brain biopsy that was negative for infectious agents but showed hypercellular tissue with scattered lymphocytes, which was concerning for low-grade glioma or reactive gliosis (Figures 3-4). Neuropathologic examination revealed anaplastic astrocytoma WHO Grade III [4]. The tissue was negative for both the 1p19q deletion and the IDH 132 mutation. The cancer progressed, and the patient passed away within four weeks of diagnosis.  

## Discussion

### Epidemiology

Anaplastic astrocytoma (AA) is a WHO Grade III brain glioma. The annual incidence is 0.44 per 100,000 [1].

### Clinical features

Symptoms are caused by local brain invasion and increased intracranial pressure. Headache and seizures are the most common symptoms, occurring in about 50% of the cases [6]. Other symptoms, including memory loss, motor weakness, language deficit, and cognitive and personality changes, occur in 20% of cases [7].

### Differential diagnosis

Intracranial lesions in adults are most often due to metastatic disease or infection, especially in the presence of multifocal brain masses. Primary brain glioma should be on the differential of even multifocal brain masses due to their aggressive and possibly lethal nature. A stereotactic brain biopsy is imperative for early and conclusive diagnosis. Other causes of multiple brain masses include multiple sclerosis, toxoplasmosis, cerebral abscess and cystic lesions, tuberculoma, neurocysticercosis, and primary CNS lymphoma [8-9]. The most common brain tumors in humans are gliomas, which are divided into four grades by WHO classification: Grades I and II are less malignant gliomas and Grades III (anaplastic astrocytoma) and IV (glioblastoma) are the more malignant gliomas [4]. Causes of a solitary brain mass include anaplastic astrocytoma (Grade III) and glioblastoma (Grade IV), both of which are malignant astrocytomas and infiltrating tumors [3]. Anaplastic astrocytomas, however, have a better prognosis than glioblastomas and are more likely of the two to respond to treatment [7].

### Diagnosis

Diagnosis is accomplished by first obtaining an image of the brain lesions and then performing a stereotactic brain biopsy to obtain a tissue sample for microscopy evaluation. AA most often involves the white matter of the cerebral hemispheres but may rarely occur in other regions of the central nervous system (CNS). AA usually presents as a solitary lesion with slow-growing tumors. Our patient presented in a highly unusual fashion with multiple rapidly growing lesions involving both cerebral lobes and the cerebellum. To reach a definitive diagnosis, a stereotactic brain biopsy with evaluation of the histopathology of the tissue sample are the gold standard [7].

### Investigations

For patients who present with neurological deficits, a CT of the head needs to be ordered to assess for brain masses, evidence of a stroke, or new intracranial hemorrhage. A CT of the chest, abdomen, and pelvis is needed to assess for the existence of a primary tumor in the case of malignancy with suspicion of metastatic lesions to the brain. A CT scan of the chest, abdomen, and pelvis can also help evaluate for signs of infection in the case of an infectious etiology leading to brain lesions. Bronchoscopy with bronchoalveolar lavage can be performed to collect biopsies of the lung to assess cytology for malignancy evaluation and culture to evaluate for infectious etiology. A stereotactic brain biopsy is imperative for early and conclusive diagnosis. The distinct histologic features of an anaplastic astrocytoma include copious pleomorphic astrocytes with an indication of mitosis (Figures 3-4) [7]. Gene mutations, such as the 1p19q deletion and IDH132 mutation, affect the prognosis as those with both the 1p19q deletion and IDH132 mutation have the best prognosis, while those without either mutation will have a poorer prognosis, as seen in the case presented above [1-2, 5].

### Prognosis

The presence of the 1p19q deletion and IDH132 mutation predicts a better prognosis [1-2]. The patient presented in this case report had neither the 1p19q deletion nor the IDH132 mutation and, therefore, carried a poor prognosis. Overall, anaplastic astrocytoma (Grade III glioma) has a two-year survival rate of approximately 50%, which is significantly higher than that for glioblastomas (Grade IV glioma) at < 20% [4].

### Treatment

Gene mutations, such as the 1p19q and IDH132, affect the prognosis and should be tested to guide therapy [1-2, 5]. Solitary brain masses are usually treated with surgical resection, while multiple brain masses are treated with radiotherapy and chemotherapy [10]. Treatment for anaplastic astrocytoma includes surgical debulking, followed by radiation and then adjuvant chemotherapy with temozolomide (TZM) [5, 7, 10]. Anaplastic astrocytoma tumors often recur and are less responsive to treatment interventions [7].

## Conclusions

Although most primary brain gliomas present as a single brain mass that is slow-growing, it is important to recognize that this is not always the case; it is possible to have multiple rapidly progressive brain masses with primary brain glioma.

Imaging can often be misleading, and stereotactic brain biopsy with histopathology remains the gold standard for reaching a diagnosis and, therefore, should be pursued early in the workup.

## References

[1] van den Bent MJ, Dubbink HJ, Marie Y (2010). IDH1 and IDH2 mutations are prognostic but not predictive for outcome in anaplastic oligodendroglial tumors: a report of the European Organization for Research and Treatment of Cancer Brain Tumor Group. Clin Cancer Res.

[2] Jiang H, Ren X, Cui X (2013). 1p/19q codeletion and IDH1/2 mutation identified a subtype of anaplastic oligoastrocytomas with prognosis as favorable as anaplastic oligodendrogliomas. Neuro Oncol.

[3] Rao SA, Srinivasan S, Patric IR (2014). A 16-gene signature distinguishes anaplastic astrocytoma from glioblastoma. PLoS One.

[4] Sonoda Y, Ozawa T, Aldape KD (2001). Akt pathway activation converts anaplastic astrocytoma to glioblastoma multiforme in a human astrocyte model of glioma. Cancer Res.

[5] Grimm SA, Chamberlain MC (2016). Anaplastic astrocytoma. CNS Oncol.

[6] Russo M, Villani V, Taga A (2017). Headache as a presenting symptom of glioma: A cross-sectional study. Cephalalgia.

[7] See SJ, Gilbert MR (2004). Anaplastic astrocytoma: diagnosis, prognosis, and management. Semin Oncol.

[8] Berger JR (2003). Mass lesions of the brain in AIDS: the dilemmas of distinguishing toxoplasmosis from primary CNS lymphoma. AJNR Am J Neuroradiol.

[9] Poptani H, Gupta RK, Jain VK (1995). Cystic intracranial mass lesions: Possible role of in vivo MR spectroscopy in its differential diagnosis. Magn Reson Imaging.

[10] Wu J, Zou T, Bai HX (2017). Comparison of chemoradiotherapy with radiotherapy alone for "biopsy only" anaplastic astrocytoma. Oncotarget.

